# Comment on “Isolating Polaritonic 2D-IR Transmission Spectra”

**DOI:** 10.1021/acs.jpclett.2c01264

**Published:** 2023-02-02

**Authors:** Blake S. Simpkins, Zimo Yang, Adam D. Dunkelberger, Igor Vurgaftman, Jeffrey C. Owrutsky, Wei Xiong

**Affiliations:** † US Naval Research Laboratory, Washington, DC20375, United States; ‡ Department of Chemistry and Biochemistry, University of California San Diego, La Jolla, California92093, United States

## Abstract

This Viewpoint responds to the analysis of 2D IR spectra of vibration cavity polaritons in the study reported in *The Journal of Physical Chemistry Letters* (Duan et al. **2021**, 12, 11406). That report analyzed 2D IR spectra of strongly coupled molecules, such as W(CO)_6_ and nitroprusside anion, based on subtracting a background signal generated by polariton filtered free space signals. They assigned the resulting response as being due to excited polaritons. We point out in this Viewpoint that virtually all of the response can be properly reproduced using the physics of transmission through an etalon containing a material modeled with a complex dielectric function describing the ground- and excited-state absorber populations. Furthermore, such a coupled system cannot be described as a scaled sum of the bare molecular and cavity responses.

A recent paper by Duan et al., “Isolating Polaritonic 2D-IR Transmission Spectra”,^1^ has challenged our analysis and interpretation of 2D IR spectra of vibration-cavity polaritons and proposed a new method of analyzing them. The main conclusions from ref (1) are that the 2D IR spectra of cavity-coupled molecular vibrations contain large contributions from “uncoupled” molecules whose optical response is filtered by the polariton transmission spectrum and that these contributions can be identified and removed to yield a signal corresponding solely to polaritonic excited-state transitions. While ref (1) appropriately acknowledges the existence of dark modes and highlights the role of spectral diffusion (an important phenomenon ignored by previous works), their treatment leads to a conclusion fundamentally different from that which our groups have recently published, but with little comparison to all but the most recent of those works.^2−6^ In light of the keen interest and current excitement surrounding the science of molecular polaritons, we believe it is critical to share our viewpoint with the aim of improving the interpretation of ultrafast polariton nonlinear optical responses.

In this Viewpoint, we make the following points regarding the transient response of systems under vibrational strong coupling (VSC). First, any description of the system’s optical response must include the fundamental physics of transmission through a cavity modulated by an absorptive medium, which can be calculated analytically using the classical transfer matrix model.^2−9^ Treating the nonlinear response of the coupled system as a separable linear combination of the response of the cavity and the molecular subsystems, as is done in ref (1), can lead to spectral artifacts incorrectly attributed to polariton response. Second, we employ two methods to show that the filtering and subtraction method described in ref (1) yields a background that is far too weak and of the wrong spectral shape to account for a significant fraction of the 2D IR polariton response. Third, we point out that “uncoupled” molecules located near modal nodes cannot make a significant contribution to the observed signal because these molecules are “uncoupled” only to the extent they do *not* influence the optical spectra. To the extent they *do* couple to the optical field, their response is nearly indistinguishable from molecules positioned elsewhere in the cavity. Lastly, we also show the conclusion in ref (1) is incompatible with the cavity length dependence of the transient response. We conclude with our overall view of the transient spectroscopy of VSC systems and discuss the areas in which we believe ref (1) does offer important insight.

## VSC Transient Response Is Primarily Rabi Splitting Contraction and Weakly Coupled Excited-State Absorption

**Figure 1:**
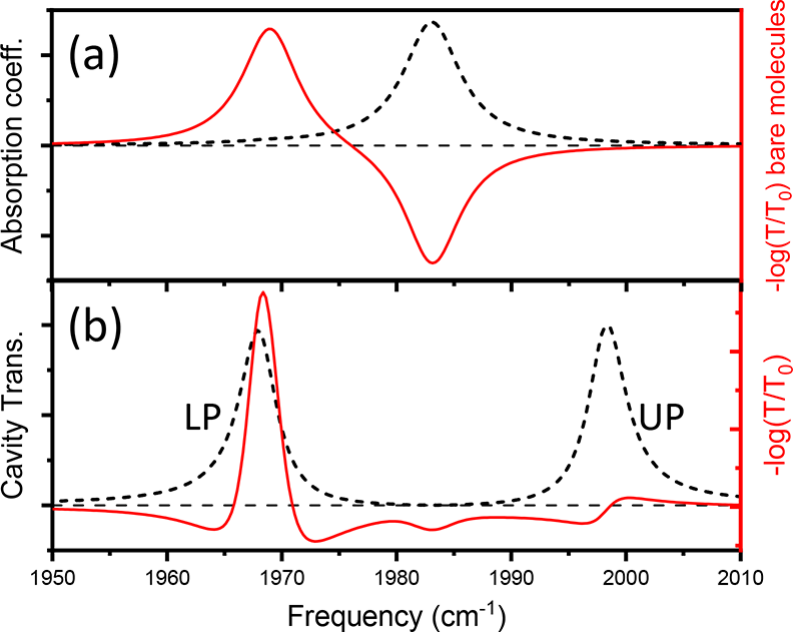
Predicted linear and transient response of the carbonyl absorption of ∼15 mM W(CO)_6_ in hexane. (a) Absorption coefficient (black) and differential transmission with 1% excitation (red). (b) Steady-state (black) and transient (red) response of same solution under strong cavity coupling. Strong coupling results in polaritons near 1970 and 2000 cm^–1^ (labeled LP and UP). Excited-state absorption of reservoir molecules is enhanced due to its coincidence with the LP mode. Reduced ground-state population causes derivative feature at UP.

To put this viewpoint into context, we will describe our treatment of the static and transient IR (and 2D IR) response, and although we will focus on the late-time response (i.e., after polariton dephasing), understanding the origins of this response led by population states is critical in qualifying any approach to subtracting it. We use a simple Fresnel description (see the Supporting Information) which accounts for the physics of transmission through an absorbing medium bounded by reflective surfaces.^9^ This classical treatment is strictly valid for the probe transmission only when early time nonlinear interactions between polaritons have decayed. Our groups have compared classical and quantum treatments of cavity transmission in this same system and demonstrated that they are equivalent in their essential features as long as anharmonicity of the transition energy and strength (referred to as *mechanical* and *electrical* anharmonicity, respectively) are included.^4,7^ The normalized static absorption and differential transmission response for W(CO)_6_ in hexane in free space are calculated in Figure 1a. When 1% of the molecules are excited to the first excited vibrational state, the transient response (red curve) exhibits oppositely signed peaks of approximately equal magnitude (assuming harmonic oscillator transition dipole scaling) that correspond to an excited-state absorption (*v* = 1 to 2; ω_12_ = 1968 cm^–1^) and a bleach and stimulated emission of the fundamental vibrational transition (*v* = 0 to 1 and 1 to 0; ω_01_ = 1983 cm^–1^).

The static and transient response of this same solution placed between lossless mirrors (*R* = 0.94) is shown in Figure 1b (15 mM solution resulting in Rabi splitting of ∼30 cm^–1^). We note that this analytical approach is completely agnostic to how absorbers come to reside in the first excited state but accurately predicts the system response under this condition. The excitation shifts the UP to lower frequency because there are fewer molecules at *v* = 0 contributing to the Rabi splitting (recall Ω, where Ω is Rabi splitting and *N*_o_ is the ground-state population.) This so-called Rabi splitting contraction results in a derivative line shape appearing near the UP-transition frequency (ω_UP_), a well-recognized effect that has been observed and described many times in both vibrational^2−4,7,10−14^ and excitonic^15−18^ strong coupling. In addition, the excited-state population yields a strong absorptive signal that occurs near the LP transition frequency (ω_LP_) due to a coincidence between ω_LP_ and ω_12_ (i.e., Ω/2 is close to the anharmonic shift). We emphasize that this feature occurs due to dark *reservoir* states (i.e., molecules in the first excited state) whose lifetime is essentially close to that observed for bare molecules outside the cavity.^2,3,11^ However, even though this signal originates from reservoir molecules in their first excited state, the spectral details of this response, and in fact those of all observed spectral features, arise from the *interaction between the absorbing medium and the cavity*.

**Figure 2:**
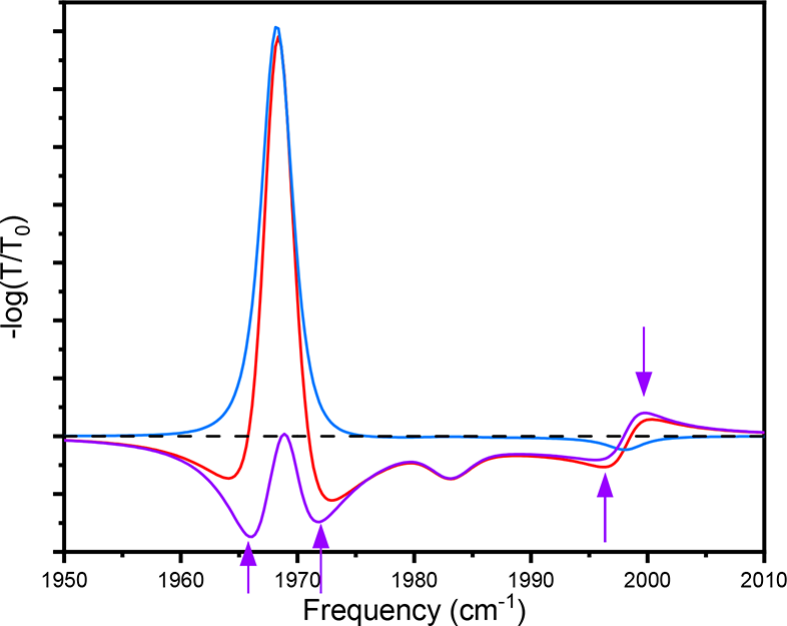
Background signal proposed by ref (1) (blue) calculated as product of bare molecule excited-state spectrum and strongly coupled spectrum. Transient response (red) for comparison to proposed background signal. Application of background subtraction (blue subtracted from red) to yield purported polariton-only response (purple).

The authors of ref (1), on the other hand, assert that the observed transient response of reservoir modes is separable from other cavity signals, that they can be removed via a linear subtraction, and that this leaves only signatures of polaritonic excited-state transitions. We reproduce their procedure via calculation by multiplying the excited-state response of free-space molecules (Figure 1a, red) by the coupled spectrum (i.e., using the polariton transmission as a “filter”; Figure 1b, black) to arrive at a scaled “background” signal (Figure 2, blue) that may be subtracted from the transient response of the entire coupled system. The proposition is that this background signal can be scaled to the size of the transient polariton responses and then subtracted from the cavity-coupled transient spectrum to yield a response attributed solely to excited-state polaritons. Such an approach relies on several conceptions that are unsound in polariton physics, which we will now discuss.

## The Optical Responses of Cavity and Molecular Systems Are Not Linearly Separable

The approach of ref (1) treats the optical responses of the cavity and molecular system as linearly separable, describing the transmission spectrum of the coupled system as a filter through which one can excite and probe the molecules and polaritons. In reality, the molecules in the cavity can be measured by the transmitted probe pulse only insofar as they participate in the formation of the transmissive polariton modes. Therefore, any attempt to separate the bare-molecule response from the polariton response via a scaled subtraction of bare molecular response is fundamentally flawed. To illustrate this in a concrete example, we refer to Figure 2. Notably, there are many features left in the spectrum after applying the proposed background subtraction (see purple arrows). Recall that in this simulation, there are no excited polariton states. It explicitly only includes changing the distribution of vibrational population of the ground- and reservoir excited-state molecules within the cavity. If subtracting the polariton-filtered free-space response effectively removed all contributions from reservoir molecules, then the purple curve would be an entirely null signal since the coupled reservoir model contains no polariton excited-state transitions. Instead, the purple trace shows numerous features that would erroneously be identified as polaritonic excited states. The filtering approach does not account for polariton contraction and only part of the excited-state absorption, so it is inaccurate to ascribe these residuals to excited polariton populations. Critically, the derivative feature near ω_UP_ (∼2000 cm^–1^) is virtually unchanged by the polariton-filtered free-space background subtraction, leading the authors of ref (1) to incorrectly assign the feature to ESA and GSB on the polariton manifold. We reiterate that this feature can be simply explained by reduced ground-state population and associated Rabi splitting contraction, without any contribution from an excited polariton. This simpler explanation is further supported by observed lifetimes that are consistent with that of the bare molecule.

Polariton-filtered free-space background subtraction fails because it does not account for the effects that modified ground- and excited-state populations have on polariton formation in the presence of molecular anharmonicities.^7^ Further evidence that the details of molecular ensemble populations and the cavity transmission are not separable can be found in numerous examples of ultrafast modulation of Rabi splitting in the collective coupling regime.^2−4,7,10−13,15−18^ We note that in ref (11), a subset of the authors here treated the experimentally determined coupled-reservoir response as a background which we subtracted from the early time transient polaritonic responses. Our subtraction approach is distinctly different from that of ref (1), because the experimental coupled-reservoir responses are directly measured through the coupled system, and therefore, their impact on the entire cavity transmission spectrum is preserved.

## The Filtered Signal Background Is Small Due to the Low Polariton Transmission

**Figure 3:**
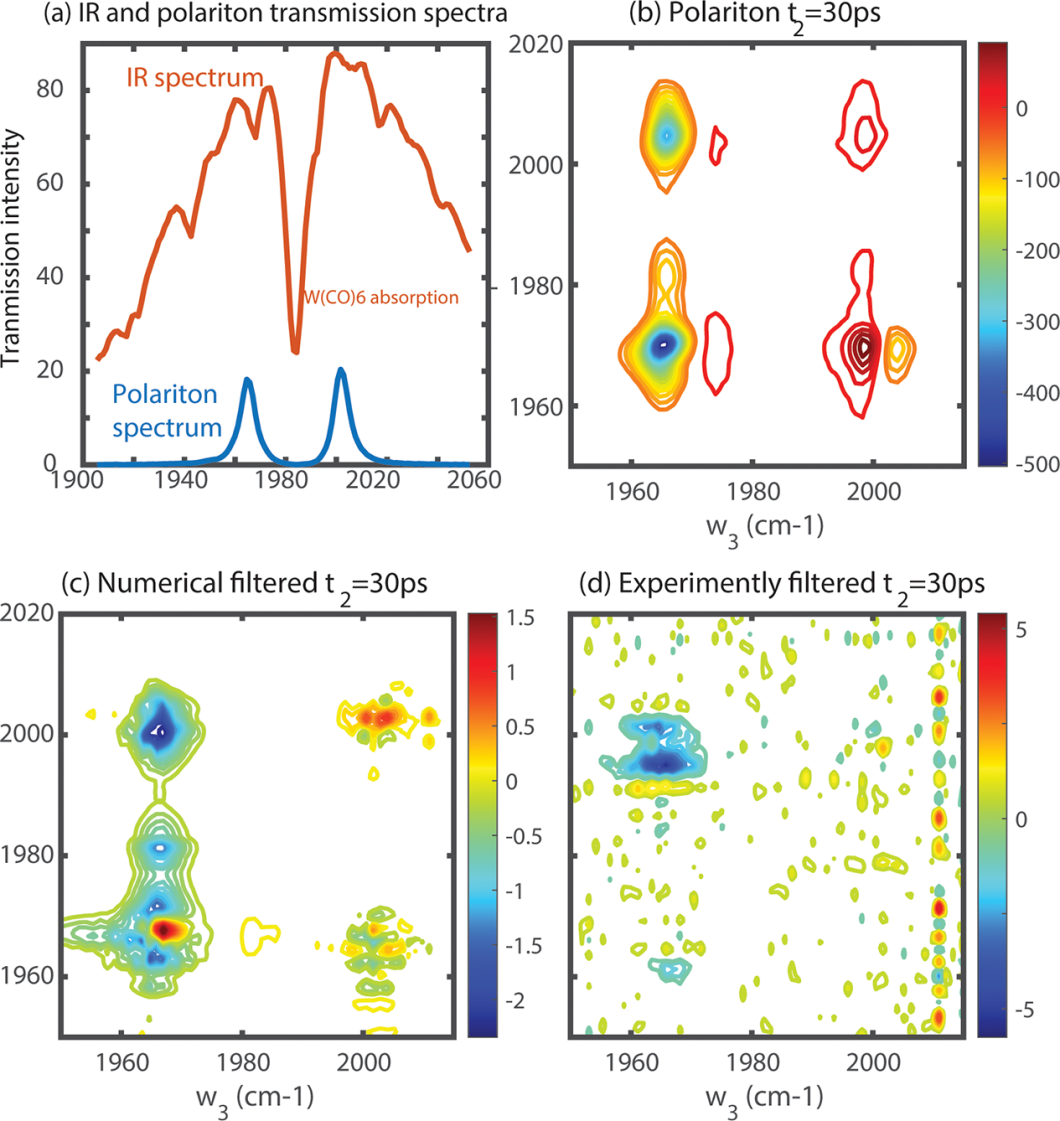
Polariton filter effect. (a) IR spectrum with W(CO)_6_ in hexane absorption and IR polariton spectrum. The polariton significantly reduces the amount of IR transmitted and interacts with the sample as a “filter”. Comparison of 2D IR spectra of (b) W(CO)_6_:hexane polariton system, (c) W(CO)_6_:hexane filtered numerically by polariton transmission spectra, and (d) W(CO)_6_:hexane filtered experimentally by polariton transmission spectra. All spectra were scaled to ensure they are compared under the same incidence IR power. ω_1_ and ω_3_ are the pump and probe frequencies, respectively. The filtered spectra from uncoupled molecules show much smaller intensity.

We will now show that the magnitude of the so-called background signal described in ref (1) is quite small, requiring a scaling of ∼300× to satisfy the authors’ condition that inhomogeneous broadening be removed by the subtraction. We have followed the treatment in ref (1) to examine the purported background signal in cavity-coupled W(CO)_6_ in hexane using both numerical and experimental filtering. First, we show the transmission spectrum of W(CO)_6_:hexane outside of the cavity and the resulting cavity-coupled response (Figure 3a). The late-time 2D IR response (Figure 3b) shows typical features found and reported in the literature.^4,7,11−13^ Next, we generate the proposed filtered background numerically, and then experimentally. In the numerical approach, following ref (1), we multiply the free-space 2D IR response of W(CO)_6_:hexane by the linear transmission spectrum of the coupled system along the pump and probe spectral axes (ω_1_ and ω_3_, respectively) to arrive at the *numerically filtered* data in Figure 3c (see Figure S2 too). We note that the 2D IR response of W(CO)_6_ in free space and under cavity coupling are collected under the same incoming IR pulse energy, so the results properly compare the signal amplitudes. Just as reported in ref (1), the filtered spectrum is >200× weaker than the original 2D IR spectra of vibration-polaritons (Figure 3b,c).

In the experimental filtering approach, both pump and probe signals were structured to follow the polariton linear spectral shape and intensity by using a pulse shaper to tailor the *pump* pulse^11,12^ and inserting a strongly coupled cavity to act as a filter in the *probe* path. The 2D IR response of such a physically filtered experiment for free-space W(CO)_6_ is shown in Figure 3d. The spectrum is properly scaled to account for pulse energy differences (see the Supporting Information). Again, we see the experimentally filtered spectra showed an extremely low signal amplitude along with fewer features and more noise. The experimentally filtered spectrum shows a large peak at [ω_UP_, ω_LP_] but with other features missing, possibly due to the small signal-to-noise ratio and the small pulse energy failing to excite higher vibrational levels of the molecules.

Overall, both the numerically and experimentally filtered spectra have much lower intensities than the actual polariton spectrum because of the substantially reduced IR pulse powers. Our results agree with the results from ref (1) in the sense that very large multiplicative factors (∼300) are needed to raise the polariton spectrum filtered background signal to the level observed in the polariton 2D IR spectra.

## Effects of Molecule Location in the Cavity

**Figure 4:**
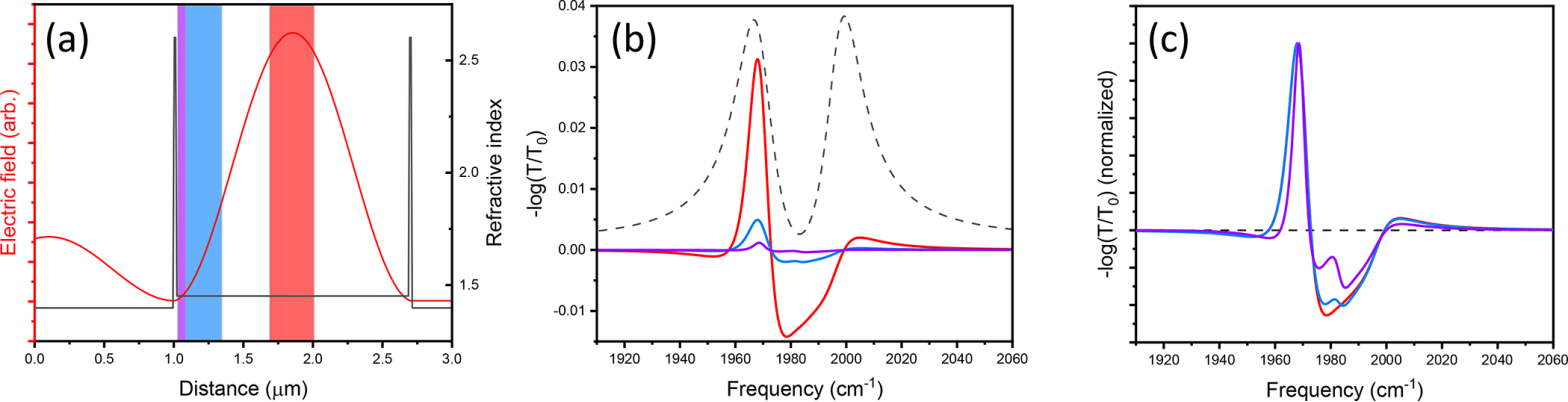
Simulated response of first-order cavity under various excitation conditions. (a) The cavity field profile and colored bars to highlight regions where excitation is localized for subsequent calculations. (b) The differential transmission for systems with 1% of the total population excited but confining the excited molecules to 1/5th of the cavity volume localized at either the antinode (red) or node (blue) of the cavity mode or an even narrower region, 1/20th of cavity, at the node (purple). The dashed line shows the empty cavity transmission. (c) The transient spectra from panel b are normalized and show an essentially identical qualitative response. These results indicate that the signal ascribed to bare molecular excitations (i.e., “background” response) likely originates from high field regions within the cavity.

We next show evidence that the large scaling factors used are indeed inappropriate and that the spectrum of excited molecules does not match a filtered free-space response, even for molecules residing at the cavity nodes, which the authors of ref (1) assert are the origin for an uncoupled molecular background signal. We model the transient response of a first-order cavity under 1% excitation, but we spatially localize all of the excited molecules at different positions within the cavity mode. The mode profile is shown in Figure 4a. We compare the response for molecular excitation centered at the antinode (indicated by the red band) to excitation localized near the mirror face (this would correspond to “uncoupled” molecules described by ref (1) and is shown as a blue band). We also calculate an example of a much narrower region of excitation located at the mirror (purple) to model more extreme localization at a node. In all cases, the total excitation level, averaged over the entire cavity, is fixed at 1% (e.g., 5% excitation localized to 1/5th the cavity length corresponds to 1% excitation averaged over the entire cavity). While the contribution that a given molecule makes to the overall coupling depends on its position within the cavity,^19^ so does the magnitude of the observed optical response. This is shown in the results of Figure 4b where the magnitude of the transient signal is maximized when molecular excitation is localized at the cavity field maximum. However, when normalized (Figure 4c), the response is qualitatively the same aside from small differences near the central frequency (∼1980 cm^–1^). This difference is due to enhanced signals occurring when excited molecules are located at the cavity antinode. This effect is further emphasized for the first-order cavity examined here. There are two important points here: (1) the signal contribution from regions near the nodes is much weaker than contributions from the antinodes and therefore cannot be a source of significant background, because near nodes, the strength of the external laser field is also close to zero, which makes the third-order nonlinear signal small, and (2) more importantly, regardless of where the signal originates, its spectrum is that of a coupled reservoir, as illustrated by the normalized transient spectra in Figure 4c. In other words, molecules at the nodes do not contribute a distinct filtered free-space response that must be removed but exhibit the coupled reservoir response, only with a smaller intensity.

## Pathlength Dependence of Polariton and Molecular Signals

To provide additional evidence that the responses of filtered background and coupled systems are different, we evaluated how the ultrafast dynamics of both systems depend on sample path length. Basically, we applied the numerical filter to *t*_2_-dependent 2D IR of W(CO)_6_ in free space and then plotted the *t*_2_ dependent integrated intensity of the [ω_LP_, ω_LP_] peak of both filtered background and 2D IR polaritonic spectra. We chose the [ω_LP_, ω_LP_] peaks because they were the ones most influenced by the approach used in ref (1).

**Figure 5:**
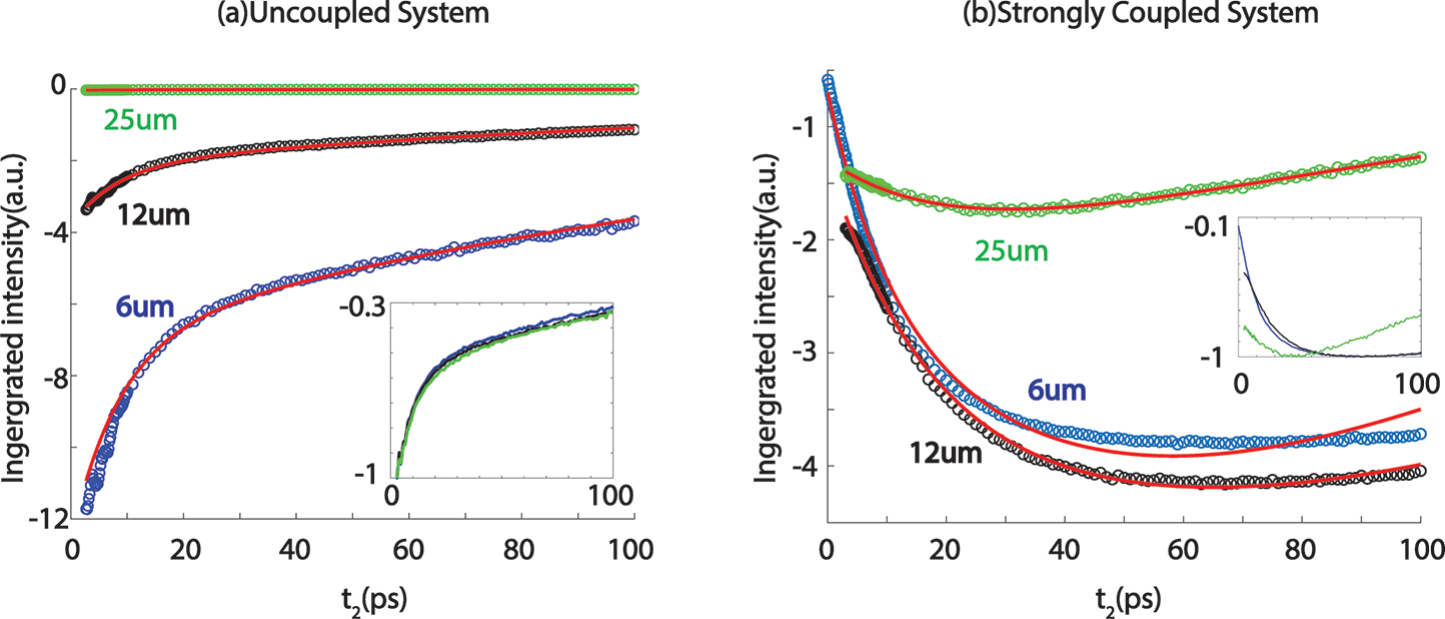
Dynamics dependence on sample path length. (a) The ν = 1 → 2 vibrational dynamics of W(CO)6 in hexane outside of the cavity exhibit clearly different signal magnitudes, but when normalized (inset), they demonstrate identical dynamics. (b) The dynamics of ν = 1 → 2 transitions of the dark reservoir modes at ω_LP_ show a qualitatively different dependence on cavity thickness, suggesting that the dynamics of strongly coupled systems are not dominated by the free space molecular dynamics.

Figure 5 shows the integrated dynamics at different thickness of both samples. The dynamics of the samples differ, as does their dependence on sample thickness. For the cavity-filtered free-space molecules, the intensity of the transient signal increases drastically as the sample thickness decreases (Figure 5a). This is a consequence of the numerical filtering applied since the shorter cavities will have lower-order modes resonant with the molecular transition. The lower-order modes are broader, leading to more transmission and therefore larger signals relative to thicker samples. However, once normalized (inset of Figure 5a), all three samples have identical dynamics. Using a simple kinetic model (details in the Supporting Information), we found the lifetime of the *v* = 1 state of dark modes to be ∼150 ps, agreeing with the literature.^2^ In contrast, the dynamics of the cavity system appears cavity thickness-dependent (Figure 5b) with the 25 μm path length sample showing the fastest decay (shown in the normalized dynamics in Figure 5b inset). If, as ref (1) asserts, the filtered background dominates the dynamics at [ω_LP_, ω_LP_], then, for the 6 μm thick sample which has the strongest nonlinear signa of uncoupled molecles, the response of the uncoupled molecules should dominate such that the signal approaches those observed for the uncoupled molecules with decreasing cavity length (Figure 5a). This trend is not observed, suggesting that uncoupled molecular dynamics do not dominate the dynamic features in the cavity systems. Instead, the different dynamics observed for various cavity lengths are related to varying levels of excitation in higher-lying excited states. Using the same kinetic model, we found that as the cavity thickness increases (from 6 to 25 μm), the relative population of the second excited state over the first excited state drops from ∼20 times to 3 times, while outside the cavity, the second excited state was not populated. Thus, the cavity thickness can modify the relaxation pathways from the polariton to the dark reservoir mode level (see the Supporting Information). This effect likely depends on the polariton linewidth, which will be the subject of future studies.^20^

## Conclusion

Based on the evidence presented above, our view of the nonlinear spectroscopy of cavity-coupled vibrational polaritons is that the reservoir response in the cavity does not have the same spectral form as a filtered free-space response, and therefore, the direct subtraction proposed in ref (1) is ineffective. In ref (1), the polariton 2D IR responses were calculated based on the third-order molecular response function used in 2D IR spectra of molecular systems. The polariton eigen-energies were determined based on a Hamiltonian describing the strong coupling between seven molecules and cavity modes. While this approach has merit, the actual polariton systems studied comprise ∼10^10^ molecules, for which other groups have calculated the polariton energies are close to harmonic.^21^ Without including any polaritonic contributions, harmonic or anharmonic, the derivative shape seen in 2D IR spectra of polaritons at the long time limit can be described by Rabi splitting contraction rather than the typical GSB, stimulated emission and excited-state absorption separated by an anharmonic potential of molecular vibrations in free space.

While important questions remain regarding early time spectral features, we believe the long-lived 2D IR responses in ref (1), which were assigned to polariton excited-state features, in actual fact arise purely from reservoir excitation. Here we must point out that there is disagreement between even the authors contributing to this Viewpoint on the nature of the nonlinear response at times longer than the total dephasing time of the polariton modes. In one view, assigning any long-lived component to polaritons is unphysical, as this time scale is much longer than the weighted sum of inverse lifetimes of the participating modes. Although there is one exception to this expectation (discussed below),^11^ in virtually all of the work in this field, responses measured after the decay of the Rabi oscillations can be fully described by excitation of reservoir molecules to their excited states, which occurs via the dephasing of polaritons. Furthermore, these late-time excitations decay with similar lifetime as free-space molecular excitations, supporting the interpretation that they are weakly coupled reservoir states.^2,4^ We argue that the inhomogeneity in ref (1) occurs because the reservoir excited-state response probed in the LP–LP region is only weakly coupled to the cavity and the narrowing effect seen in strong coupling may not apply.

Several authors of this Viewpoint hold a different view, having recently reported features in the 2D IR of cavity-coupled nitroprusside that were difficult to explain by reservoir excitation or a polariton bleach.^11^ In ref (11), we subtracted the coupled response from the reservoir population to isolate small features that we assigned to long-lived polaritonic populations. For the reasons outlined above, we find this subtraction to be more appropriate than subtracting the filtered free-space spectrum. These features last several picoseconds longer than the polariton dephasing time and had peak positions reasonably well predicted by an anharmonic Hamiltonian used in ref (1), albeit with some important mismatches. In this work, a subset of authors argued that the molecular character of the polariton modes might lead to lifetimes longer than the total dephasing time.

It is in reference to this work on nitroprusside^11^ that we think ref (1) has particularly useful insight. Spectral diffusion is an important effect that has been ignored in these systems, and ref (1) rightly turns attention to this problem. One of the arguments posed in ref (1) was that the spectral diffusion dynamics of the lower diagonal peaks strongly agree with those of the uncoupled system and the much larger inhomogeneity of their molecular system (compared to molecular systems in hexane in the original polariton 2D IR studies) places it at the border of the strong coupling regime, which results in increased overlap between the polariton and dark states and an increased role of spectral diffusion. Duan et al. show that the filtered background approach can qualitatively reproduce the majority of the early time features we observed. This is an important result that we did not consider in ref (11) and is a reasonable assignment of the features we observed. We would expect, however, that the system is better described by considering the transmission through a cavity with a subset of the inhomogeneous distribution of oscillators excited. That is to say, a more rigorous model would include the inhomogeneity of the band in the coupled-reservoir model. Such a treatment might resolve the few discrepancies between the spectra in refs (1) and (11), and we are actively pursuing including inhomogeneity in the coupled reservoir model.

We close by reiterating the primary point that any model of the optical response of a cavity-coupled species must recognize that cavity transmission spectra are shaped by the absorptive media it contains and that one cannot directly subtract free-space molecular response, regardless of scaling.

## Supporting Information Available

Classical modeling of transmission through an absorptive media, calibration of 2D IR excitation intensities, and fitting results of the polariton dynamics (PDF)
