# Supplementary material for: Comment on “Isolating Polaritonic 2D-IR Transmission Spectra”
Source: J Phys Chem Lett. 2023 Feb 2;14(4):983–8. doi: 10.1021/acs.jpclett.2c01264 (PMC9900631; doi:10.1021/acs.jpclett.2c01264)
Supplement: Supplementary file 1 — jz2c01264_si_001.pdf [file jz2c01264_si_001.pdf]

Supporting Information for Comment on "Isolating Polaritonic 2D-IR Transmission Spectra"

Blake S. Simpkins,[1] Zimo Yang,[2] Adam D. Dunkelberger,[1] Igor Vurgaftman,[1] Jeffrey C. Owrutsky,[1] and Wei Xiong,[2]

1. US Naval Research Laboratory, Washington, DC 20375
2. Department of Chemistry and Biochemistry, University of California San Diego, La Jolla, California 92093, USA

Classical Modeling of Transmission through an Absorptive Media

The transmittance spectra were calculated according to the following expression for transmission through a Fabry-Pérot cavity filled with an absorptive media.

$$T_{cav}(\nu) = \frac{(1-R)^2 e^{-\alpha L}}{1+R^2 e^{-2\alpha L}+2Re^{-\alpha L}\cos(4\pi n L\nu+2\varphi)} \quad \text{(S1)}$$

$$n = \sqrt{\frac{\varepsilon_1+\sqrt{\varepsilon_1^2+\varepsilon_2^2}}{2}} \quad \text{(S2)}$$

$$\alpha = 4\pi\nu\sqrt{\frac{-\varepsilon_1+\sqrt{\varepsilon_1^2+\varepsilon_2^2}}{2}} \quad \text{(S3)}$$

$$\varepsilon_1 = n_{bg}^2 + \sum_i \frac{A_i(\nu_i^2-\nu^2)}{(\nu_i^2-\nu^2)^2+(\Gamma_i\nu)^2} \quad \text{(S4)}$$

$$\varepsilon_2 = \sum_i \frac{A_i\Gamma_i\nu}{(\nu_i^2-\nu^2)^2+(\Gamma_i\nu)^2} \quad \text{(S5)}$$

In this expression, ν is frequency, R mirror reflectivity, α the absorption coefficient, L cavity length, n refractive index, and φ the phase shift upon reflection. Both α and n are frequency-dependent and are described by a Lorentzian oscillator model shown as Eq. S2 and S3 with ε_1 and ε_2 defined in Eq. S4 and S5. To account for distinct ground and excited-state populations, ε_1 and ε_2 can consist of multiple states corresponding to elements in the series over *i*. Calculation of transient spectral response can be done by varying the amplitude factors, A_i in ε_1 and ε_2 to mimic population changes.

Below, we showed the spectral cut of 2D IR at $\omega_1 = \omega_{dark}$. It shows derivative feature on the UP side and a large absorptive feature on the LP side. Clearly, this cannot be modeled by the filtered approach (blue trace in Figure 2). Thus even the dark mode spectra cut is not due to the spectral filter effect.

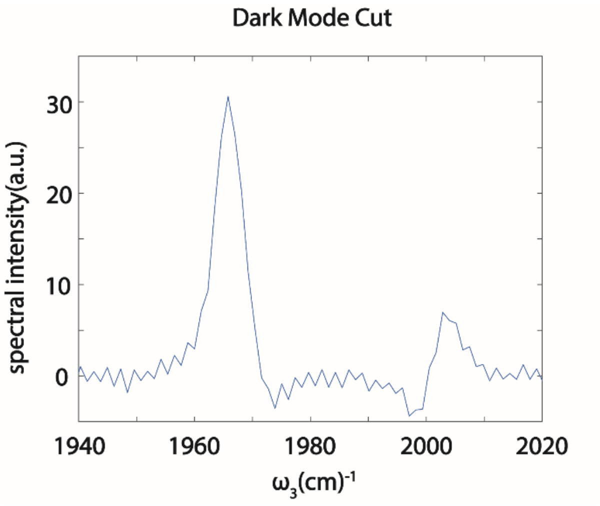

Figure 1. Spectral cut at $\omega_1 = \omega_{dark}$ of Figure 3(b)

Calibration of 2D IR Excitation Intensities

To properly determine any 2D IR contribution from uncoupled molecules but interacted by the filtered IR spectra through polariton samples, it is necessary conduct these measurements and the regular 2D IR on polariton samples under the same laser excitation conditions. We start by deriving how to determine the filtered 2D IR spectra from uncoupled molecules. Assuming there is a 2D IR response from the uncoupled molecules due to interaction with the pump and probe electromagnetic fields, its signal in the time domain should be:

$$S_t = E_{2DIR}(t; t_2, t_1) \propto P^{(3)}(t; t_2, t_1) \propto \int_0^\infty dt_3' \int_0^\infty dt_2' \int_0^\infty dt_1' \sum_n R_n(t_3', t_2', t_1') E_3(t - t_3') E_2(t + t_2 - t_3' - t_2') E_1(t + t_2 + t_1 - t_3' - t_2' - t_1') \quad \text{(S6)}$$

which can be simplified to

$$E_{2DIR}(t; t_2, t_1) \propto \{R * E_1 * E_2 * E_3\}(t; t_2, t_1) \quad . \quad \text{(S7)}$$

This is the convolution of the three pulses with the response function of the system. Because the 2D IR signals are presented in the frequency domain, which involves the Fourier transform of $E_{2DIR}(t; t_2, t_1)$ $(FT(E_{2DIR}(t; t_2, t_1)))$ amplified by the local oscillator,

$$E_{LO}(\omega) = FT\{E_3\} = E_3(\omega) \text{ (self-heterodyned)} \quad \text{(S8)}$$

which yields

$$S_f = E_{2DIR}(\omega_3; t_2, \omega_1) = FT\{E_{2DIR}(t; t_2, t_1)\} * E_{LO}(\omega) \propto FT\{R * E_1 * E_2 * E_3\}(t, t_2, t_1) * E_{LO}(\omega) = FT\{R\} \cdot FT\{E_3\} \cdot FT\{E_2\} \cdot FT\{E_1\} * E_{LO}(\omega) = FT\{R\} * E_3^2(\omega) * E_1(\omega) * E_2(\omega) = FT\{R\} * I_{pump,filtered}(\omega) * I_{probe,filtered}(\omega) \quad \text{(S9)}$$

In the derivation above, we applied the convolution theorem and the self-heterodyne local oscillator field, i.e.,

$$E_{LO}(\omega) = FT\{E_3\} = E_3(\omega) \quad \text{(S10)}$$

where the product of E_1 and E_2 comprise the filtered pump pulse,

$$E_1(\omega) * E_2(\omega) = I_{pump,filtered}(\omega) \tag{S11}$$

and the product of E_3 and ELO comprise the filtered probe pulse,

$$E_3(\omega) * E_{LO}(\omega) = I_{probe,filtered}(\omega) \tag{S12}$$

Here, $FT\{R\}$ is the 2D IR spectrum under impulsive limit assumption, and thereby can be represented by the broadband pump probe.

When the system interacts with the filtered pump/probe pulse, it produces the signal

$$S_f \propto FT\{R\} * I_{pump,filtered} I_{probe,filtered} = FT\{R\} * I_{pump} I_{probe} * F^2 \tag{S13}$$

Where $I_{pump/probe,filtered}$ are the intensities of the corresponding lasers, and $I_{pump/probe}$ are the incoming laser intensities before passing through polaritons. $I_{pump/probe,\ filtered}=I_{pump/probe}*F$. It is straight forward to rewrite Eq. S13 into

$$S_f \propto S_{molecule} * F^2 \tag{S14}$$

Where $S_{molecule}$ is the 2D IR response of molecular samples when interacting with broadband pulses without polariton filtering, and it can be experimentally measured. Practically, to avoid saturating the detector, the IR probe intensity is attenuated when measuring $S_{molecule}$, so that $I_{probe,an} = I_{probe} * An$, where An is the attenuation factor. Thus, the 2D IR signal of molecules after attenuating the IR probe beam is $S_{molecule,an}=S_{molecule}*An$, and

$$S_f \propto S_{molecule,an}/An * F^2 \tag{S15}$$

The last step is to determine F, which

$$F = \frac{I_{probe,filtered}}{I_{probe}} = \frac{I_{probe,filtered}}{I_{probe,an}/An} = F_{an} * An \tag{S16}$$

where F_{an} is the ratio between the experimentally measured polariton filtered IR probe spectrum and the attenuated full broadband IR spectra. Combining Eqs. S15 and 16, we get

$$S_f = S_{molecular,an} * {F_{an}}^2 * An = S_{molecular,an} * F_{an} * F \tag{S17}$$

Where $S_{molecular,an}$ and F_{an} are both experimentally measured and the attenuation factor, An = 0.21 (Fig.S1).

We obtain the experimentally filtered 2DIR S_{Ef} by inserting a polariton sample on the probe path to filter the probe beam, and use the pulse shaper to create pump pulses whose spectral lineshape matches the polariton spectrum. Because the shaped pump power and the pump power after the polariton samples are different, we need to scale the experimentally measured filtered spectra S_{Ef}^{raw} by a scale factor SF, in order to obtain the S_{Ef} measured under the same condition as the regular 2D IR of polaritons.

$$S_{Ef} = S_{Ef}^{raw} * SF \tag{S18}$$

And $SF = \frac{I_{pump,filtered}}{I_{shaped}}$, where both intensities are measured experimentally.

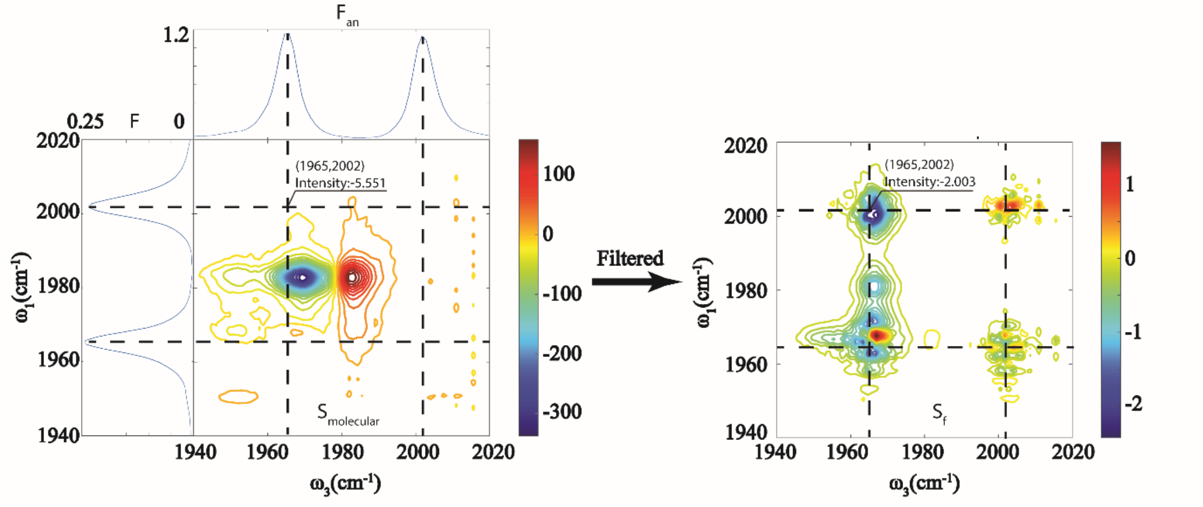

Figure S2. The molecular spectra and corresponding pump and probe filters for the filtered spectra. Following Eq.S17, the broadband 2D IR spectrum ($S_{molecular,an}$) of the uncoupled molecules are multiplied by the polariton linear transmission spectra along the ω1 (F), and ω3 (F_{an}) axes, respectively, to generate the filtered spectra (S_f) . The noted signal intensity of $S_{molecular,an}$ has the same spectral location (1965,2002) of the peak in the filter spectra (noted by *), using Eq.S17, by multiplying the intensity of (1965,2002) of $S_{molecular,an}$ by $F*F_{an}$, the peak intensity at the same position of S_f can be obtained.

Fitting results of the polariton dynamics.

We fitted the LP dynamics using the following kinetic model, and the fitted results are shown in Fig.5. Because it is known that at long time delay, the LP dynamics reflect the 1-2 transition of the dark reservoir modes, we used the kinetic model below to describe its dynamics.

$P_2'[t] = -k_{21} * P_2[t],$

$P_1'[t] = k_{21} * P_2[t] - k_{10} * P_1[t]$

$P_0'[t] = k_{10} * P_1[t]$

$P_2[0] = A_2, P_1[0] = A_1, P_0[0] = A_0$ (S19)

P_N stands for the number of populations of *Nth excited* state. k_{ab} stands for the transfer rate between excited levels *a* and *b*. In the model, we also assumed that $k_{21} = 2 * k_{10}$. The resultant solution for P_1, including the rotational diffusion dynamics, is

$$P_1[t] = [(2 * A_1 + A_2) * e^{-k_{10}*t} - 2 * A_1 * e^{-2*k_{10}*t}] * (\frac{1}{9} + \frac{4}{45} e^{-6*D*t}) \qquad \text{(S20)}$$

Where D is the rotational diffusion constant.

The result is summarized in Table S1. k_{10} become slightly faster under VSC comparing to outside cavity, which remain to be further investigated. The biggest effect is that the relative population between 2nd and 1st excited states (P_2/P_1) change when the thickness are tuned, with the thinner spacers more effectively exciting the 2nd excited state. This phenomenon could be related to the hot vibrational dynamics reported by some of the authors.

Table S1. Fitting results of Figure 5.

	6um	12um	25um	Outside of cavity
P_2/P_1	18.6	9.7	2.6	0
$T_{10} = 1/k_{10}$ (ps)	112	130	107	153
